# Evaluation of methods to assign cell type labels to cell clusters from single-cell RNA-sequencing data

**DOI:** 10.12688/f1000research.18490.3

**Published:** 2019-10-14

**Authors:** J. Javier Diaz-Mejia, Elaine C. Meng, Alexander R. Pico, Sonya A. MacParland, Troy Ketela, Trevor J. Pugh, Gary D. Bader, John H. Morris

**Affiliations:** 1 Princess Margaret Cancer Centre, University Health Network, Toronto, ON, M5G 2M9, Canada; 2 The Donnelly Centre, University of Toronto, Toronto, ON, M5S 3E1, Canada; 3 Department of Pharmaceutical Chemistry, University of California San Francisco, San Francisco, CA, 94143, USA; 4 Gladstone Institutes, San Francisco, CA, 94158, USA; 5 Multi-Organ Transplant Program, Toronto General Hospital Research Institute, Toronto, ON, M5G 2C4, Canada; 6 Department of Immunology, University of Toronto, Toronto, ON, M5S 1A8, Canada; 7 Department of Laboratory Medicine and Pathobiology, University of Toronto, Toronto, ON, M5G 1L7, Canada; 8 Department of Medical Biophysics, University of Toronto, Toronto, ON, M5G 1L7, Canada; 9 Ontario Institute for Cancer Research, Toronto, ON, M5G 0A3, Canada; 10 Department of Molecular Genetics, University of Toronto, Toronto, ON, M5G 1A8, Canada

**Keywords:** single cell, RNA-seq, scRNA-seq, bioinformatics, benchmark, evaluation, labeling, cell type

## Abstract

**Background:** Identification of cell type subpopulations from complex cell mixtures using single-cell RNA-sequencing (scRNA-seq) data includes automated steps from normalization to cell clustering. However, assigning cell type labels to cell clusters is often conducted manually, resulting in limited documentation, low reproducibility and uncontrolled vocabularies. This is partially due to the scarcity of reference cell type signatures and because some methods support limited cell type signatures.

**Methods:** In this study, we benchmarked five methods representing first-generation enrichment analysis (ORA), second-generation approaches (GSEA and GSVA), machine learning tools (CIBERSORT) and network-based neighbor voting (METANEIGHBOR), for the task of assigning cell type labels to cell clusters from scRNA-seq data. We used five scRNA-seq datasets: human liver, 11 Tabula Muris mouse tissues, two human peripheral blood mononuclear cell datasets, and mouse retinal neurons, for which reference cell type signatures were available. The datasets span Drop-seq, 10X Chromium and Seq-Well technologies and range in size from ~3,700 to ~68,000 cells.

**Results:** Our results show that, in general, all five methods perform well in the task as evaluated by receiver operating characteristic curve analysis (average area under the curve (AUC) = 0.91, sd = 0.06), whereas precision-recall analyses show a wide variation depending on the method and dataset (average AUC = 0.53, sd = 0.24). We observed an influence of the number of genes in cell type signatures on performance, with smaller signatures leading more frequently to incorrect results.

**Conclusions:** GSVA was the overall top performer and was more robust in cell type signature subsampling simulations, although different methods performed well using different datasets. METANEIGHBOR and GSVA were the fastest methods. CIBERSORT and METANEIGHBOR were more influenced than the other methods by analyses including only expected cell types. We provide an extensible framework that can be used to evaluate other methods and datasets at https://github.com/jdime/scRNAseq_cell_cluster_labeling.

## Introduction

During the last five years a number of single-cell sequencing technologies have been developed to identify cell subpopulations from complex cell mixtures ( Bakken *et al*., 2017). For instance, recent advances in single-cell RNA-sequencing (scRNA-seq) enable the simultaneous measurement of expression levels of thousands of genes across thousands of individual cells. The resulting expression matrices of genes by cells are used to identify cell subpopulations with characteristic gene expression profiles (i.e. cell types).

A typical computational pipeline to process scRNA-seq data involves: i) quality control of sequencing reads, ii) mapping reads against a reference transcriptome, iii) normalization of mapped reads to correct batch effects and remove contaminants, iv) data dimensionality reduction with principal component analysis or other approaches, v) clustering of cells using the reduced dimensionality representation, vi) detection of genes differentially expressed between clusters, vii) visualization of cell clusters in t-SNE or similar methods, and viii) assignment of cell type labels to cell clusters. A number of computational tools, including Cell Ranger ( Zheng *et al*., 2017a) and Seurat ( Butler *et al*., 2018), support automation of steps i to vii ( Duò *et al*., 2018; Freytag *et al*., 2018; Innes & Bader, 2019). However, assignment of cell type labels to cell clusters is still conducted manually by most researchers. The typical procedure involves manual inspection of the genes expressed in a cluster, combined with a detailed literature search to identify if any of those genes are known gene expression markers for cell types of interest. This manual approach has several caveats, including limited documentation and low reproducibility of cell type gene marker selection, use of uncontrolled and non-standard vocabularies for cell type labels, and it is time-consuming. For these reasons, computational tools that enable researchers to systematically, reproducibly and quickly assign cell type labels to cell clusters derived from scRNA-seq experiments are needed.

**Figure 1.**
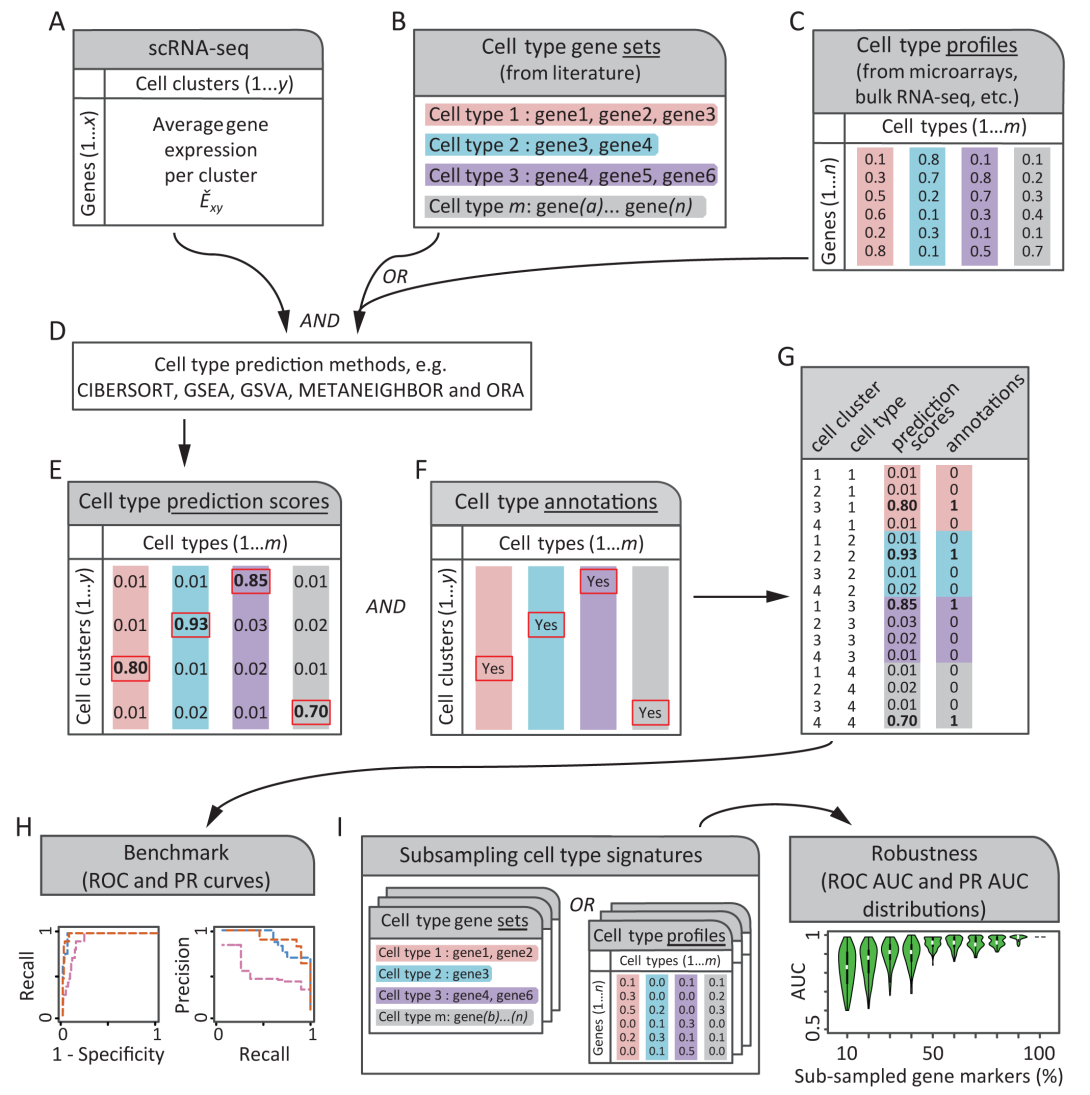
Schematic of a process to benchmark automated cell type prediction methods. Two inputs are needed by automated cell type prediction methods ( **A**– **C**). ( **A**) a matrix with the average expression of each gene *x* for each cell cluster *y* ( *Ě _xy_*). ( **B**, **C**) cell type gene marker signatures can be provided as either gene sets (lists of gene identifiers, B) or numeric gene expression profiles ( **C**). ( **D**) Gene sets can be manually compiled from literature and are used for methods like GSEA, GSVA or ORA, whereas gene-expression profiles are measurements from microarrays, bulk- or single-cell RNA-sequencing (scRNA-seq) experiments and are used by methods like CIBERSORT and METANEIGHBOR. ( **E**) Automated cell type prediction methods produce a matrix of cell type prediction scores for each cell cluster. ( **F**) Some authors of scRNA-seq studies assign cell type labels manually to cell clusters using local expertise or orthogonal experiments such as fluorescence activated cell sorting. These annotations can be used as a gold standard to benchmark automated cell type predictions. ( **G**) Cell type prediction scores (from **E**) for cell clusters are concatenated into a single vector and known cell cluster annotations (from **F**) are added. The resulting matrix is used to assess the performance of cell type prediction methods by receiver operating characteristic (ROC) curve and precision-recall (PR) curve analyses varying over the prediction scores for all cell clusters in a dataset ( **H**). ( **I**) Robustness of cell type prediction methods can be analysed by gradually subsampling gene markers from cell type gene expression signatures ( **B** or **C**) and repeating procedures of ( **D**– **H**) to obtain distributions of the area under the curve (AUC) for ROC (ROC AUC) and PR (PR AUC) curves, which are shown as violin plots. We hypothesized that some prediction methods are more robust than others to the proportion of gene markers subsampled from cell type gene expression signatures.

In this study we analysed each of five scRNA-seq datasets with five computational methods that can be used to assign cell type labels to cell clusters based on known gene expression marker lists. The datasets include human liver cells ( MacParland *et al*., 2018); mouse retinal neurons ( Shekhar *et al*., 2016b); the Tabula Muris mouse cell atlas data ( Tabula Muris Consortium *et al*., 2018a), which encompasses 20 tissues of which we used 11 for which cell type signatures were available ( Tabula Muris Consortium, 2018b); and human peripheral blood mononuclear cells (PBMCs) mapped using two technologies: 10X Chromium ( Zheng *et al*., 2017a) and Seq-Well ( Gierahn *et al*., 2017a) ( Table 1). We chose these five datasets primarily because they provided expert curated known cell type marker gene lists and cell cluster annotations that we could use as gold standards. The five methods analysed were: CIBERSORT ( Newman *et al*., 2015b), GSEA ( Subramanian *et al*., 2005), GSVA ( Hänzelmann *et al*., 2013), METANEIGHBOR ( Crow *et al*., 2018) and ORA ( Fisher, 1935; Goeman & Bühlmann, 2007) ( Table 2). We chose these five methods to represent different categories of algorithms, ranging from first-generation enrichment analysis (ORA) to second-generation approaches (GSEA and GSVA), machine learning tools (CIBERSORT) and network-based neighbor voting approaches (METANEIGHBOR). Although ORA and GSEA were not originally developed to process RNA-seq data, they have been extensively used in transcriptomic studies for gene set enrichment analyses. GSVA was developed to analyse microarray and bulk RNA-seq data. CIBERSORT was developed to estimate abundances of cell types in mixed cell populations from bulk RNA-seq data, and METANEIGHBOR was developed to characterize replicability of scRNA-seq samples. We adapted all five methods to assign cell type labels to cell clusters from scRNA-seq data based on known sets of cell type marker genes. We evaluated these methods using two types of inputs: a matrix with the average expression of each gene *x* from all the cells in each cell cluster *y* ( *Ě _xy_*) from scRNA-seq measurements, which we assume corresponds to the profile of a cell type or state ( Figure 1A), and known cell type signatures, represented as gene sets ( Figure 1B) or continuous gene expression profiles ( Figure 1C).

**Table 1.** scRNA-seq datasets used in this study.

Dataset Name	Description of scRNA-seq dataset	Number of genes in *Ě _xy_*	Number of cells	Number of cell clusters	Number of cell type signatures	Reference
Liver	10X Chromium sample from liver cells from five human donors	20,007	8,444	20	10	( MacParland *et al*., 2018)
Retinal neurons	Drop-seq sample from retinal bipolar neurons from healthy mice	13,166	27,499	18	15	( Shekhar *et al*., 2016b)
Tabula Muris	10X Chromium samples from 11 out of 20 mouse tissues with cell type signatures, or 6 out of those 11, with signatures for three or more cell types	18,300	55,505	130	53	( Tabula Muris Consortium *et al*., 2018a)
PBMCs-10X	10X Chromium sample from peripheral blood mononuclear cells from a human donor	17,786	68,579	7	22 or 6	( Zheng *et al*., 2017a)
PBMCs-SeqWell	Seq-Well sample from peripheral blood mononuclear cells from human	6,713	3,693	6	22 or 6	( Gierahn *et al*., 2017a)

**Table 2.** Cell cluster labeling methods compared in this study.

Acronym	Version	Name	Language	Reference
CIBERSORT	1.01	Cell type Identification by Estimating Relative Subsets of RNA Transcripts	R and Java	( Newman *et al*., 2015b)
GSEA	3.0	Gene Set Enrichment Analysis	Java	( Subramanian *et al*., 2005)
GSVA	1.30	Gene Set Variation Analysis	R	( Hänzelmann *et al*., 2013)
METANEIGHBOR	1.3.1	Meta-analysis via neighbor voting	R	( Crow *et al*., 2018)
ORA	R( 3.5.1)	Over- representation Analysis	R	( Fisher, 1935; Goeman & Bühlmann, 2007)

## Methods

### Generation of cell cluster average gene expression matrices ( *Ě _xy_*)

For all datasets, the *Ě _xy_* matrix was obtained from cell cluster scRNA-seq measurements with AverageExpression(use.raw = T) from Seurat v2 ( Butler *et al*., 2018). For the liver dataset ( MacParland *et al*., 2018) (NCBI GEO: GSE115469) we followed the authors’ reported cell cluster assignments and applied AverageExpression() to compute the average expression profile for all cells in each cluster. For the retinal neuron dataset ( Shekhar *et al*., 2016b) (NCBI GEO: GSE81905) the gene expression matrix and cell cluster assignments were obtained from ( Shekhar *et al*., 2016a) and processed with AverageExpression(). For the Tabula Muris dataset (NCBI GEO: GSE109774), the droplet (10X Genomics) RNA measurements were obtained from Figshare ( Tabula Muris Consortium, 2018b). File ‘annotations_droplet.csv’ was used to obtain tissue and cell cluster information, and AverageExpression() was applied to clusters from each tissue. ‘Tabula Muris 11’ includes a subset of this data containing the 11 tissues for which we could map cell type gene expression signatures into cell clusters (see below), whereas ‘Tabula Muris 6’ was a subset of 6 tissues for which the mapped signatures had at least three cell types per tissue. The list of tissues included can be found in the corresponding *Ě _xy_* matrices provided as Supplementary Information ( Diaz-Mejia *et al*., 2019a). For the PBMC-10X datasets ( Zheng *et al*., 2017a), ‘Fresh 68k PBMCs DonorA’ gene expression matrix files were obtained from 10X Genomics ( Zheng *et al*., 2017b) (NCBI Sequence Read Archive: SRX1723926). Normalization, data dimensionality reduction and cell clustering were conducted with Seurat v2 with the following functions: FilterCells(low.thresholds = 200,-Inf, high.thresholds = 0.05,10000); FindClusters(reduction.type = “pca”, dims.use = 1:10, resolution = 0.4); and AverageExpression(). For the PBMC-SeqWell datasets ( Gierahn *et al*., 2017b), the GSM2486333_PBMC.txt file with read counts was obtained from GEO dataset GSM2486333 (NCBI GEO: GSE92495). Columns with labels ‘Removed_*’ were removed and from the remaining columns, column header prefixes, like: BCELL*, NK*, CD4*, etc., were used to classify cells into clusters, and AverageExpression() was applied to each cluster.

### Collection of cell type gene expression signatures

A gene expression signature is defined as a set of genes characteristically and detectably expressed in a cell type. These are typically inferred from small-scale experiments manually identified in the literature, or by comparing the transcriptome of a given cell type against all other available cell type gene expression profiles, usually from the same experiment. The liver cell type gene set signatures were manually curated by us (author S.A.M.) and were originally used to manually annotate cell types in the liver dataset ( MacParland *et al*., 2018). We provide these gene sets on Zenodo ( Diaz-Mejia *et al*., 2019a). For the retinal neuron dataset ( Shekhar *et al*., 2016b), known cell type markers reported by the authors were used as cell type gene set signatures. For the Tabula Muris dataset, one of the consortium authors provided us with manually defined cell type gene set signatures for 11 of the 20 tissues included in the publication. The file is now available in the Tabula Muris repository ( Tabula Muris Consortium, 2018c; Tabula Muris Consortium, 2019). For the PBMC-10X and PBMC-SeqWell datasets, we used a blood cell type gene expression profile signature compiled by the CIBERSORT developers called LM22, containing 547 genes and 22 cell types ( Newman *et al*., 2015a). We also tested an alternative signature designed for RNA seq data with 17 cell types ( Monaco *et al*., 2019), and it produced similar results to the LM22 dataset (Supplementary Table S2), thus we decided to use only the LM22 for our study. For the PBMC-10X dataset, reference cell type assignments were obtained from the authors’ fluorescence-activated cell sorting (FACS)-based assignments ( Zheng *et al*., 2017c). The PBMC cell clusters we obtained with Seurat were mapped using cell barcode identifiers against the FACS assignments, and cell type names were manually matched to the LM22 signature. For the PBMC-SeqWell datasets ( Gierahn *et al*., 2017a) cell cluster prefixes from the file GSM2486333_PBMC.txt column headers were used to manually assign cell types from the LM22 matrix ( Newman *et al*., 2015a).

CIBERSORT and METANEIGHBOR require as input a cell type gene expression signature in the form of gene expression profiles including gene expression scores. For the PBMC datasets, we used the LM22 signature to evaluate these two methods in two ways. First, we used the original LM22 signature ( Newman *et al*., 2015b) with continuous valued gene expression measurements, which we called CIBERSORT ‘continuous’ and METANEIGHBOR ‘continuous’. Second, for each cell type of the LM22 signature, a value of ‘1’ was assigned to 5% of genes with highest expression values in their column or a value of ‘0’ otherwise, and we called this approach CIBERSORT ‘binary’ and METANEIGHBOR ‘binary’. The same 5% of genes was used to create cell type gene set signatures as inputs for GSEA, GSVA and ORA. For the liver dataset, we transformed the cell type gene set signature into a binary matrix of genes in rows and cell types in columns for CIBERSORT ‘binary’ and METANEIGHBOR ‘binary’. To do this, each gene included in each cell type gene set *m* was assigned a value of ‘1’ in the column corresponding to *m* in the matrix, whereas other genes absent in *m* but present in other cell type gene sets were assigned a value of ‘0’. Similarly, for the retinal neuron dataset the ‘previously known markers’ for bipolar cell types provided in Table S2 of Shekhar *et al*. (2016b) were transformed into a binary matrix of genes by cell types for CIBERSORT ‘binary’ and METANEIGHBOR ‘binary’ analyses. For the Tabula Muris datasets, we organized the cell type gene set signatures of each tissue into a binary matrix of genes in rows and tissue-cell types in columns for CIBERSORT ‘binary’ and METANEIGHBOR ‘binary’ analyses.

### Generation of subsampled cell type gene expression signatures and area under the curve (AUC) distribution violin plots

Cell type signature gene sets ( Figure 1B) were subsampled by randomly removing between 10 and ~99% of genes from each signature in increments of 10%, keeping a minimum of one gene. Each subsampling of gene sets was organized as a binary matrix of genes by cell types for CIBERSORT ‘binary’ and METANEIGHBOR ‘binary’ as indicated above. Cell type gene expression profile signatures ( Figure 1C) were subsampled in two stages: first we selected the top 5% highest expressed genes for each cell type, then we replaced the gene expression value of a random 10 to 100% of those genes from each cell type, in increments of 10%, by the minimum value of the cell type column. This resulted in subsampled gene expression profile signatures with identical size to the original profile signatures, but with values of the top highly expressed genes randomly replaced by the minimum score of each cell type. For percentage values between 10 to 100%, 1,000 subsampling replicates were generated for each cell type gene expression signature, and each replicate was processed as indicated by Figure 1D–I. Violin plots were used to show the resulting ROC and PR AUC distributions.

### Implementation of tested methods and use of enrichment metrics for ROC and PR analyses

We used five methods to score each cell cluster for each cell type. Three methods (CIBERSORT, GSVA and METANEIGHBOR) generated scores that could be directly used for ROC and PR curve analyses. For ORA and GSEA, we first transformed their cell cluster labeling P-values to a -log10 scale, so that higher values reflected higher scores of a cell cluster belonging to a given cell type and used these scores for ROC and PR curve analyses. All prediction scores for each dataset over all tested cell cluster vs. cell type pairs were concatenated into a single vector and compared to gold standard cell cluster annotations ( Figure 1G). Varying prediction score thresholds over this vector was used to plot the ROC and PR curves and obtain AUC values (using R ROCR and precrec libraries). For each prediction score threshold, all predictions above the threshold were predicted positives and these were compared to known cluster annotations to identify true and false positives, as well as true and false negatives below the score threshold, for ROC and PR curve analysis. Commands for each method were: CIBERSORT (v1.01), ‘CIBERSORT.jar -M Mixture -B signature -n 1000’; R library(GSVA) v1.30, R function gsva(); GSEA v3.0, ‘gsea-3.0.jar GseaPreranked -nperm 1000’; ORA, R function fisher.test() from R v3.5.1. For ORA, the universe of genes used was the intersection of genes present in the cell type gene expression signature and the *Ě _xy_* matrix of each dataset. For METANEIGHBOR we created a modified version of function MetaNeighborUS() from the R library(MetaNeighbor) v1.3.1, to obtain cell type prediction scores. A typical MetaNeighborUS() run uses scRNA-seq measurements from studies 1 and 2, and its output is the average ROC AUC for each pair of neighbor ROC AUC scores across 'training' and 'testing' datasets. In this study, we instead used cell clusters from one scRNA-seq dataset as the 'testing' dataset (i.e. study 1) and cell type signatures as the 'training' dataset (i.e. study 2). With the advice of one of the MetaNeighbor developers, we modified function MetaNeighborUS() source code to remove the averaging command ‘cell_NV <- (cell_NV+t(cell_NV))/2’ and compiled the library from the modified source. All methods were implemented in Java, R and Perl ( Table 2). The scripts used to run and benchmark cell type labeling methods described in this study are available on GitHub and archived at Zenodo ( Diaz-Mejia *et al*., 2019b).

### Computing time benchmark

We implemented wrapper scripts to execute each of the five methods tested, including a stopwatch to time the cell type prediction task. Other tasks, such as input and output preparation, were excluded from computing time values reported in Figure 6D and Supplementary Table 1. All computing time measurements were made using a 3.1-GHz Intel Core i5 CPU with 2 cores and 16 GB RAM. Robustness analyses were performed on the Niagara supercomputer at the SciNet HPC Consortium ( Ponce *et al*., 2019).

## Results

### Benchmark of cell cluster labeling methods

We benchmarked the performance and computing time of five cell type labeling methods: CIBERSORT, GSVA, GSEA, METANEIGHBOR and ORA ( Table 2), using average gene expression profiles of scRNA-seq cell clusters and known cell type gene expression signatures. We used five scRNA-seq datasets: liver ( MacParland *et al*., 2018), retinal neurons ( Shekhar *et al*., 2016b), the Tabula Muris mouse cell atlas ( Tabula Muris Consortium *et al*., 2018a), and two PBMC datasets obtained with 10X Chromium ( Zheng *et al*., 2017a) and Seq-Well technologies ( Gierahn *et al*., 2017a) ( Table 1). Each method used two inputs: an *Ě _xy_* matrix with the average gene expression for each cell cluster ( Figure 1A) and a cell type gene expression signature, represented as either a gene set (discrete set of genes) or a gene expression profile (vector of continuous gene expression values). For three of the five methods tested (GSVA, GSEA and ORA) we used cell type signatures in the form of gene sets ( Figure 1B), and for CIBERSORT and METANEIGHBOR we used two cell type signature representation approaches: binary and continuous. In one approach we transformed gene sets into binarized matrices and called the method variants CIBERSORT ‘binary’ and METANEIGHBOR ‘binary’. In the second approach, we used available gene expression profiles ( Figure 1C) and called the method variants CIBERSORT ‘continuous’ and METANEIGHBOR ‘continuous’.

**Figure 2.**
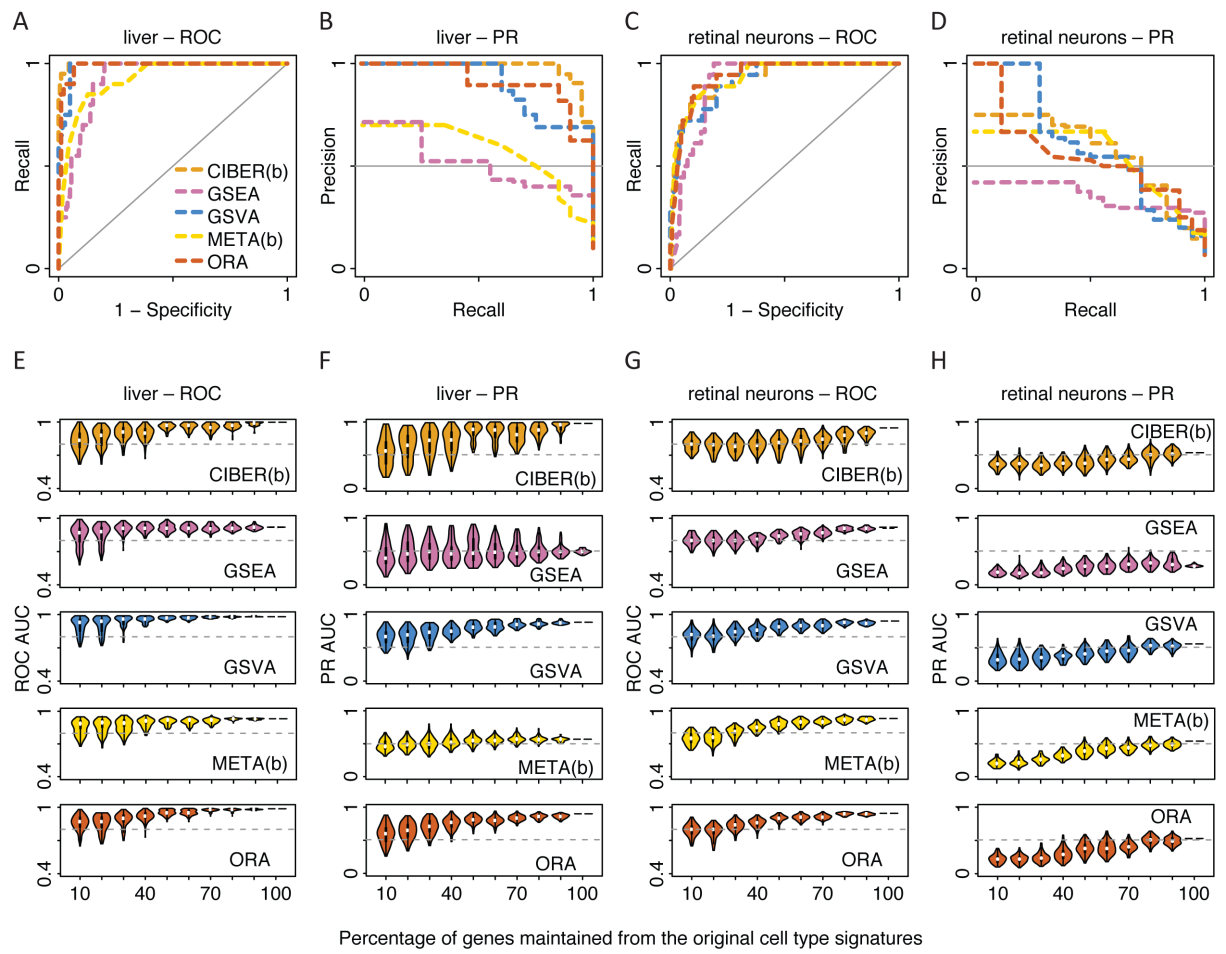
Performance and robustness analysis of cell type prediction methods using liver and retinal neuron scRNA-seq data. Receiver operating characteristic (ROC) and precision-recall (PR) curve analyses of five automated cell type prediction methods: CIBERSORT (CIBER(b)), GSEA, GSVA, METANEIGHBOR (META(b)) and ORA ( Table 2) using a human liver scRNA-seq dataset to compute ROC curve analysis ( **A**) and PR curve analysis ( **B**); using a mouse retinal neuron scRNA-seq dataset to compute ROC curve analysis ( **C**) and PR curve analysis ( **D**). The cell type gene expression signatures used for ROC and PR curve analyses for panels **A** to **D** were randomly subsampled 1,000 times, keeping 10 to 100% of genes from the original signatures each time. Automated cell type prediction was repeated for each subsample, and violin plots representing the distribution of resulting ROC AUCs and PR AUCs are shown for analyses using human liver cells to compute ROC AUC robustness ( **E**) and PR AUC robustness ( **F**), and using mouse retinal neurons to compute ROC AUC robustness ( **G**) and PR AUC robustness ( **H**).

Each method produced a matrix of cell type prediction scores ( Figure 1D, E) which was compared to manually annotated cell type gold standards ( Figure 1F, G) to conduct receiver operating characteristic (ROC) and precision-recall (PR) curve analyses ( Figure 1H). The robustness of each method was assessed by randomly subsampling 10% to 100% of the genes from cell type gene expression signatures and repeating the cell type prediction, and ROC and PR curve analyses for each subsample ( Figure 1I). In the following sections we show ROC and PR curve analyses side by side with their robustness analyses ( Figure 2 to Figure 5) and a summary of results in Figure 6.

### ROC curve analysis

**Figure 3.**
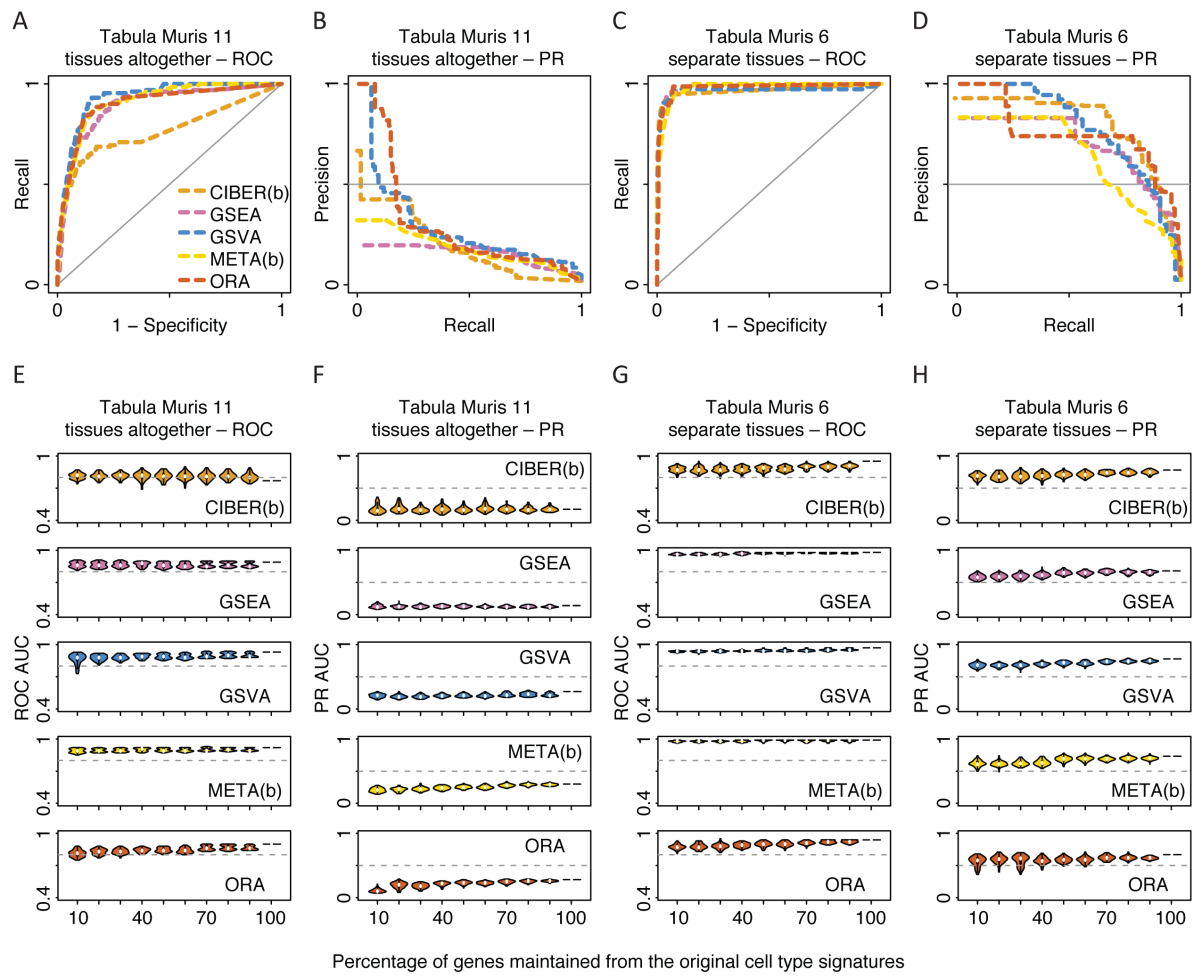
Performance and robustness analysis of cell type prediction methods using Tabula Muris scRNA-seq data. The same procedure as described in Figure 2 for ROC and PR AUCs of the liver and retinal neuron datasets was used here for the Tabula Muris dataset. Please see Figure 2 legend for details. The Tabula Muris dataset was analysed in two ways. In the first way (‘Tabula Muris 11’, panels **A**, **B**, **E** and **F**), 11 tissues whose cell type signatures and cell clusters could be mapped against each other were analysed using all cell type signatures as a single input gene set database for cell type prediction methods. In the second way (‘Tabula Muris 6’, panels **C**, **D**, **G** and **H**), the analysis was restricted to six tissues with three or more cell type signatures. In this strategy, each tissue’s cell types were predicted separately from other tissues and the results were combined afterwards to evaluate the ROC, PR and robustness of each of five automated cell type prediction methods: CIBERSORT (CIBER(b)), GSEA, GSVA, METANEIGHBOR (META(b)) and ORA.

**Figure 4.**
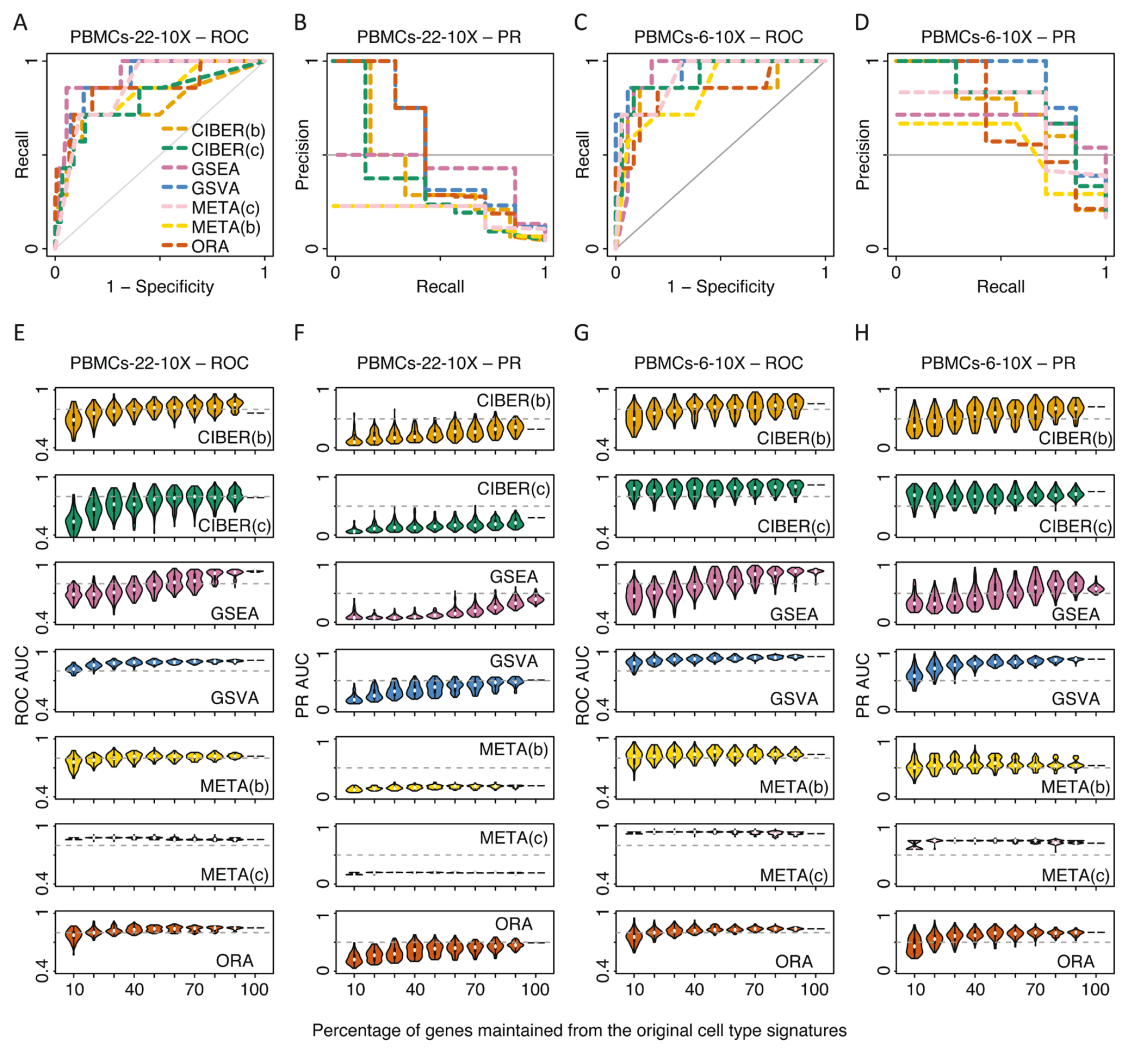
Performance and robustness analysis of cell type prediction methods using 10X PBMCs scRNA-seq data. The same procedure as described in Figure 2 for ROC and PR AUCs of the liver and retinal neuron datasets was used here for the PBMCs dataset measured with the 10X Chromium technology. Please see Figure 2 legend for details. Seven cell clusters from the 10X scRNA-seq measurements could be mapped vs. six out of 22 cell types of the PBMC LM22 matrix signatures. This dataset was analysed in two ways. In the first way (‘PBMCs-22-10X’, panels **A**, **B**, **E** and **F**), all 22 cell type signatures from the LM22 matrix were used as input for cell type prediction methods. In the second way (‘PBMCs-6-10X’,panels **C**, **D**, **G** and **H**), only the six cell types from the LM22 that could be mapped to the seven cell clusters were used as input for cell type prediction methods. For CIBERSORT and METANEIGHBOR, two approaches were used, one with the original LM22 matrix with continuous gene expression values, that we called CIBERSORT ‘continuous’ (CIBER(c)) and METANEIGHBOR ‘continuous’ (META(c)), and another with a thresholded and binarized version of the LM22 matrix, that we called CIBERSORT ‘binary’ (CIBER(b)) and METANEIGHBOR ‘binary’ (META(b)).

**Figure 5.**
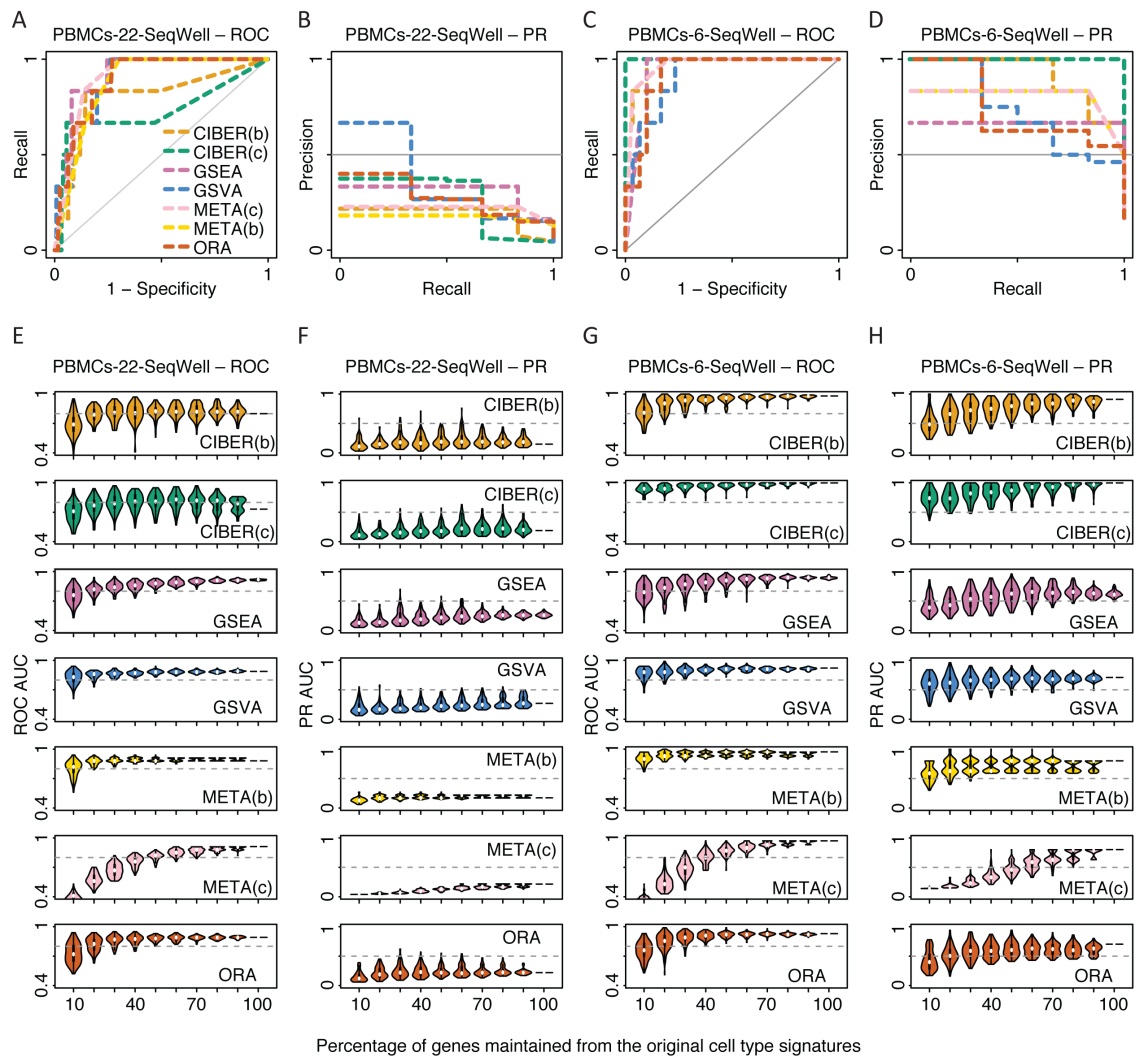
Performance and robustness analysis of cell type prediction methods using Seq-Well PBMCs scRNA-seq data. The same procedure as described in Figure 2 for ROC and PR AUCs of the liver and retinal neuron datasets was used here for the PBMC dataset measured with the Seq-Well technology. Please see Figure 2 legend for details. Six cell clusters from the Seq-Well scRNA-seq measurements could be mapped vs. six out of 22 cell types of the PBMC LM22 matrix signatures. This dataset was analysed in two ways. In the first way (‘PBMCs-22-SeqWell’, panels **A**, **B**, **E** and **F**), all 22 cell type signatures from the LM22 matrix were used as input for cell type prediction methods. In the second way (‘PBMCs-6-SeqWell’, panels **C**, **D**, **G** and **H**), only the six cell types from the LM22 that could be mapped to the six cell clusters were used as input for cell type prediction methods. For CIBERSORT and METANEIGHBOR, two approaches were used, one with the original LM22 matrix with continuous gene expression values, that we called CIBERSORT ‘continuous’ (CIBER(c)) and METANEIGHBOR ‘continuous’ (META(c)), and another with a thresholded and binarized version of the LM22 matrix, that we called CIBERSORT ‘binary’ (CIBER(b)) and METANEIGHBOR ‘binary’ (META(b)).

In general, we observed that all five methods showed high ROC AUCs for assigning cell types to all eight analysed scRNA-seq dataset variants (average ROC AUC over 40 method-data combinations = 0.91, s.d. = 0.06). The liver and retinal neuron datasets showed average ROC AUC = 0.96 and 0.93, respectively ( Figure 2A, C). The Tabula Muris dataset was analysed in two ways. In the first way, which we call ‘Tabula Muris 11’, we used data from 11 tissues for which we could find cell type signatures ( Tabula Muris Consortium, 2018b), and used their signatures collectively as a gene set database input for a single task to predict cell types across all Tabula Muris tissues (average ROC AUC = 0.88, Figure 3A). In the second way, which we call ‘Tabula Muris 6’, we restricted predictions to six tissues with three or more cell type signatures per tissue, using tissue-specific cell type signature gene set databases, and merged the prediction scores from the six tissues to evaluate performance over all those tissues (average ROC AUC = 0.97, Figure 3C). Since we observed higher ROC AUCs using ‘Tabula Muris 6’ than using ‘Tabula Muris 11’ ( Figure 3C vs. 3A), we also analysed the PBMC datasets similarly. First, we used all 22 cell type signatures from the LM22 ( Newman *et al*., 2015b) to predict cell types using PBMC cell clusters from 10X and Seq-Well. We call these approaches ‘PBMCs-22-10X’ and ‘PBMCs-22-SeqWell’ and obtained average ROC AUCs of 0.85 and 0.86, respectively ( Figure 4A, Figure 5A). Secondly, we restricted the analyses to the six cell types from the LM22 matrix that mapped to PBMC cell clusters (see Methods). We call these approaches ‘PBMCs-6-10X’ and ‘PBMCs-6-SeqWell’ and obtained average ROC AUCs of 0.89 and 0.96, respectively ( Figure 4C, Figure 5C).

**Figure 6.**
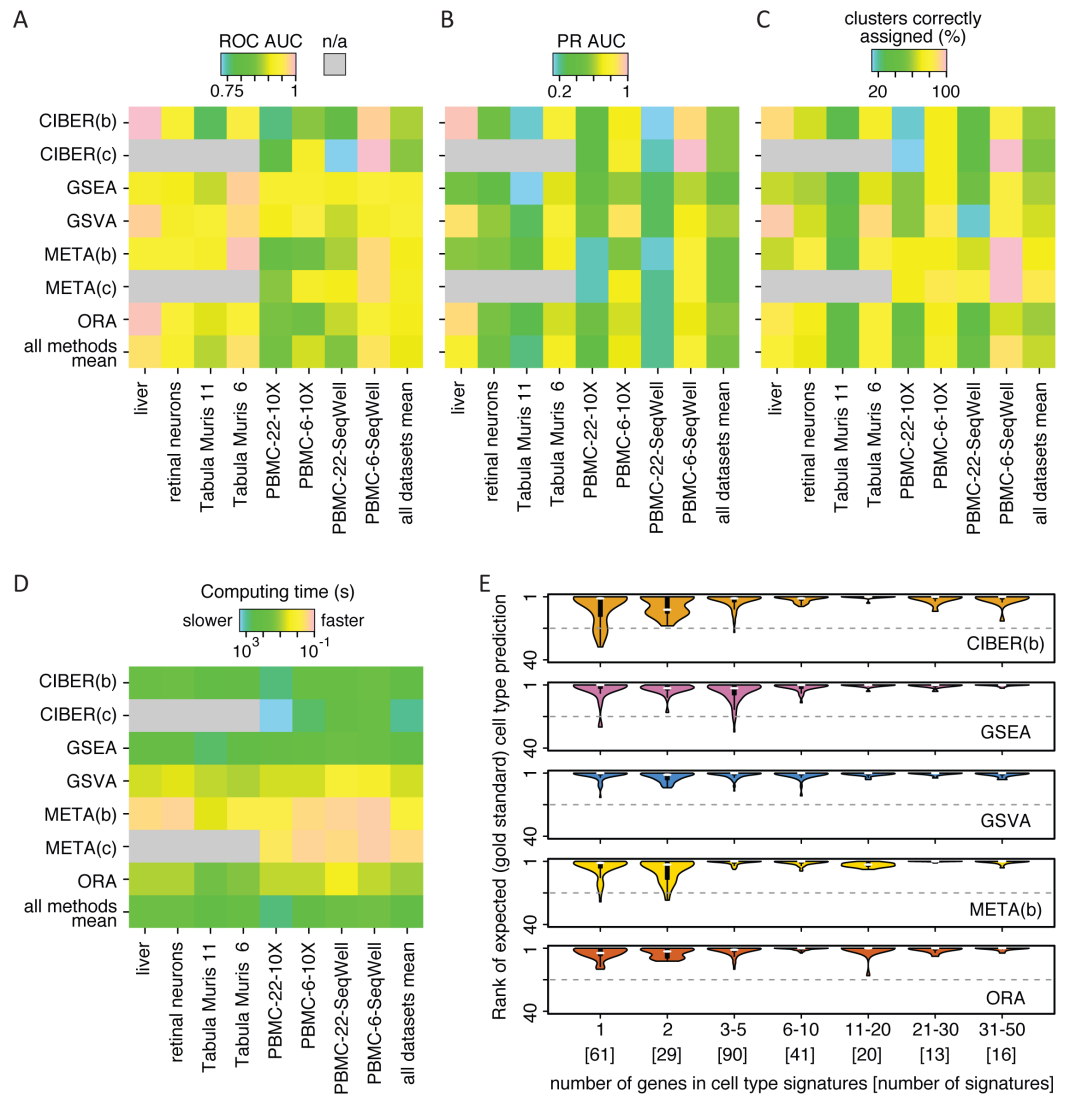
Summary of performance and computing time of cell type prediction methods using scRNA-seq data. ( **A**) A heatmap showing a summary of cell type prediction ROC AUCs for the five datasets used (x-axis), including two variants each of the Tabula Muris and PBMC datasets, for the five methods studied (y-axis), including two approaches for CIBERSORT (‘binary’ CIBER(b), and ‘continuous’ CIBER(c)) and METANEIGHBOR (‘binary’ META(b), and ‘continuous’ META(c)). The mean ROC AUCs for each method across all scRNA-seq datasets and for each dataset across all methods are provided. ( **B**) A heatmap similar to ( **A**), but showing PR AUC values. ( **C**) A heatmap showing the percentage of clusters correctly assigned by each method for each dataset. ( **D**) A heatmap showing computing times for each cell type prediction task and the mean across all scRNA-seq datasets. Actual values for panels **A** to **D** are provided in Supplementary Table 1. ( **E**) Violin plots showing the influence of the number of genes in cell type signatures on methods that use gene sets as inputs. The number of genes in the signatures is shown on the x-axis along the number of signatures with that number of genes, in brackets. The rank of the expected (gold standard) predictions is shown on the y-axes. A perfect method would show its gold standard predictions ranked as ‘1’.

In terms of ROC curve analyses, GSEA and GSVA were the top performers (average ROC AUCs = 0.93 each), closely followed by the two approaches of METANEIGHBOR and ORA (average ROC AUCs = 0.91 each), then CIBERSORT ‘binary’ (average ROC AUC = 0.88) and CIBERSORT ‘continuous’ (average ROC AUC = 0.86). Notably, the ‘binary’ approaches of CIBERSORT and METANEIGHBOR produced some of the highest performance among all tested methods for the liver (CIBERSORT ‘binary’ ROC AUC = 1, Figure 2A), retinal neuron (CIBERSORT ‘binary’ and METANEIGHBOR ‘binary’ ROC AUCs = 0.93 each, Figure 2C) and Tabula Muris datasets (METANEIGHBOR ‘binary’ ROC AUC = 0.92 using 11 tissues, and 0.99 using six tissues). In fact, the CIBERSORT and METANEIGHBOR ‘binary’ performances were comparable to those using the original LM22 matrix with continuous values, which we called CIBERSORT ‘continuous’ and METANEIGHBOR ‘continuous’ ( Figure 6A). A summary of these observations is provided in Figure 6A and Supplementary Table 1.

The analysis of ROC AUC robustness showed that in general, performance decayed as a function of removing genes from cell type gene signatures. For the liver dataset, GSVA and GSEA tolerated removal of up to 60% of genes from the liver signatures to maintain ROC AUCs ≥ 0.8, whereas CIBERSORT ‘binary’, METANEIGHBOR ‘binary’ and ORA tolerated removal of up to 50% of the genes at the same ROC AUC cutoff ( Figure 2E). For the retinal neuron dataset, GSVA, METANEIGHBOR ‘binary’ and ORA tolerated removal of up to 50% of the genes from the signature to maintain ROC AUCs ≥ 0.8, whereas GSEA and CIBERSORT ‘binary’ tolerated removal of 30% and 20%, respectively ( Figure 2G). Analysis of the Tabula Muris dataset showed that all methods were more stable to removal of genes from these signatures compared with observations for the liver, retinal neuron and PBMC datasets. The ‘Tabula Muris 6’ approach resulted in ROC AUCs slightly more robust than those using ‘Tabula Muris 11’ ( Figure 3E, G). Analysis of the PBMC datasets showed that GSVA was the method that tolerated the highest removal of genes from signatures (of up to 90%) to maintain ROC AUCs ≥ 0.8 ( Figures 4E, 4G, Figure 5E, 5G). In contrast, METANEIGHBOR ‘continuous’ was robust using the 10X PBMC dataset ( Figure 4E, G) but decayed markedly using the Seq-Well dataset ( Figure 5E, G). At the same ROC AUC cutoff = 0.8, ORA tolerated removal of up to 50% of genes and GSEA removal of 30–40% of genes for all PBMC datasets ( Figure 4E, 4G, Figure 5E, 5G). The two versions of CIBERSORT showed similar behaviours to each other, tolerating removal of up to 60% of genes in the ‘PBMC-6-SeqWell’ approach ( Figure 5G), but decayed quickly after removing only 10% of genes in the rest of the PBMC approaches ( Figures 4E, 4G, Figure 5E).

### Precision-Recall curve analysis

When benchmarking the five methods compared in this study, we classified each cell cluster positively into a single-cell type and negatively into the remaining cell types. This produced a skewed distribution with few positive predictions and many negative predictions. To address this imbalance, we used PR curve analyses in addition to ROC curve analyses. In general, the PR AUCs were smaller and more diverse (average PR AUC = 0.53, s.d. = 0.24) than the ROC AUCs (average ROC AUC = 0.91, s.d. = 0.06) ( Figure 2 to Figure 5, panels A vs. B, and C vs. D). However, when we restricted signatures to keep only cell types expected to match the input cell clusters we found that ROC AUCs increased marginally (average 1.1 times), whereas the PR AUCs increased substantially (average 3.2 times). For instance, the average PR AUC using ‘Tabula Muris 6’ was higher than that using ‘Tabula Muris 11’ (0.73 vs. 0.23, Figure 3D vs. 3B). Similarly, the average PR AUC using ‘PBMC-6-10X’ was higher than that using ‘PBMC-22-10X’ (0.69 vs. 0.34, Figure 4D vs. 4B); and the average PR AUC using ‘PBMC-6-SeqWell’ was higher than that using ‘PBMC-22-SeqWell’ (0.79 vs. 0.21, Figure 5D vs. 5B).

Some methods clearly separated from the rest using PR curve analyses. For instance, the two highest PR AUCs obtained in this study were for CIBERSORT ‘continuous’ using the ‘PBMC-6-SeqWell’ dataset (PR AUC = 1, Figure 5D) and CIBERSORT ‘binary’ using the liver dataset (PR AUC = 0.98, Figure 2B). Interestingly, CIBERSORT ‘binary’ also showed some of the lowest PR AUCs in this study, with a PR AUC = 0.17 using the ‘Tabula Muris 11’ dataset ( Figure 3B) and PR AUC = 0.15 using the ‘PBMC-22-SeqWell’ dataset ( Figure 5B). A similar behaviour was observed for METANEIGHBOR ‘binary’ and ‘continuous’ showing low PR AUCs using the ‘PBMC-22-10X’ (PR AUC = 0.19 each) and ‘PBMC-22-SeqWell’ datasets (PR AUC = 0.17 and 0.22; Figures 4B, Figure 5B), but a considerable increase using the reduced versions of the same datasets: ‘PBMC-6-10X’ (PR AUC = 0.71 for METANEIGHBOR ‘continuous’ and 0.54 for METANEIGHBOR ‘binary’) and ‘PBMC-6-SeqWell (PR AUCs = 0.8 each).

GSVA and ORA showed relatively stable PR AUCs across datasets, and GSVA was one of the methods showing the highest PR AUC using the liver, retinal neuron, ‘Tabula Muris 6’ and PBMC-10X datasets ( Figure 6B). GSEA and METANEIGHBOR ‘binary’ showed lower PR AUCs than other methods using the liver, retinal neuron and ‘Tabula Muris 11’ datasets ( Figure 2B, 2D, Figure 3B). A summary of these observations is provided in Figure 6B and Supplementary Table 1.

In terms of the PR AUC robustness analysis, in general, all five methods achieved their more robust behaviour using the Tabula Muris datasets ( Figure 3F, H). Other cases where the PR AUCs were robust include the liver ( Figure 2F) and ‘PBMC-6-SeqWell’ datasets ( Figure 5H).

GSVA was one of the methods that maintained higher PR AUC values than other methods upon removal of genes from signatures; in particular using the liver ( Figure 2F), ‘PBMC-6-10X’ ( Figure 4H) and ‘PBMC-6-SeqWell’ datasets ( Figure 5H). For instance, both GSVA and ORA tolerated removal of up to 60% of genes from the liver cell types signature to maintain PR AUCs ≥ 0.5; whereas CIBERSORT ‘binary’ tolerated removal of 50% of the genes and METANEIGHBOR ‘binary’ only 10%, using the same cutoff ( Figure 2F). METANEIGHBOR ‘continuous’ showed high PR robustness for the ‘PBMC-6-10X’ dataset ( Figure 4H), but interestingly, such behaviour was not recapitulated using the ‘PBMC-6-SeqWell’ dataset ( Figure 5H).

### Computing time benchmark

Computing times varied from 0.03s for METANEIGHBOR ‘continuous’, processing the ‘PBMC-6-SeqWell’ dataset, to 9,330s (2.6 hours) for CIBERSORT ‘continuous’, processing the ‘PBMC-22-10X’ dataset ( Figure 6D and Supplementary Table 1). For all five datasets, METANEIGHBOR ‘continuous’ was the fastest method, with times between 0.03 and 0.11s, closely followed by METANEIGHBOR ‘binary’, with times between 0.4 and 1.77s. GSVA ranked third (0.4 to 4.74s), followed by ORA (1 to 28s). GSEA was 1 to 3 orders of magnitude slower than the preceding methods (48 to 1255s). Finally, the slowest methods were CIBERSORT ‘binary’ (46 to 1,522s) and CIBERSORT ‘continuous’ (75 to 9,330s).

The size of the cell type gene expression signatures used for CIBERSORT influenced the speed of classification. For example, for the analysis of the PBMC datasets with CIBERSORT ‘continuous’ we used the original LM22 signature with 547 genes, whereas the thresholded binary matrix used for CIBERSORT ‘binary’ had 248 genes. CIBERSORT ‘continuous’ took 1.3 to 6 times longer than CIBERSORT ‘binary’ without much difference in performance ( Figure 6C and Supplementary Table 1).

### Influence of number of genes in signatures on method performance

We evaluated how the number of genes in cell type signatures affected the performance of the five tested methods. As shown in Figure 6E, all methods tended to rank positive gold standards as top hits (i.e. the greater the number of genes in cell type signatures, the greater the chances that a method correctly predicts a cell type). All methods tended to have mispredictions (ranks > 1) using cell type signatures of only one or two genes. Methods like GSEA and GSVA showed a marked improvement when the cell type signatures had 11 genes or more compared with <11 genes, whereas METANEIGHBOR ‘binary’ improved considerably when signatures had three or more genes, compared with one or two genes. CIBERSORT ‘binary’ and ORA showed a partial improvement when signatures had six or more genes, but they had peaks of mispredictions at 11–20 genes (ORA) and 21–50 genes (CIBERSORT ‘binary’).

## Discussion

The size and volume of scRNA-seq datasets are continually increasing. While most data processing is automated, cell type labeling of cell clusters is still conducted manually by most researchers. This is in part due to a scarcity of reference cell type gene expression signatures and also because most methods to address this challenge are only available via web servers supporting limited number of cell types ( Alavi *et al*., 2018; Alquicira-Hernandez *et al*., 2018), making it difficult for users to adapt them for their needs. In this study we used five scRNA-seq datasets to benchmark five methods that can address these challenges. Although three of the five tested methods (GSEA, GSVA and ORA) were not explicitly developed to identify cell types, their extensive use in gene set enrichment tasks and their wide portability motivated us to test them as cell type classifiers. METANEIGHBOR was developed to analyse scRNA-seq datasets and can be adapted to predict cell types. CIBERSORT is implemented both as a webserver and a local command line software package that can be freely licensed for six months by academic researchers, enabling us to benchmark it with relatively low programmatic effort.

Our results show that for the five scRNA-seq datasets used, all five tested methods achieved good performance by ROC curve analyses. However, ROC curves tend to overestimate performance when the ratio of positive to negative predictions is highly skewed. For this reason, we also conducted PR curve analyses. The PR curve analyses showed more variation in the performance of methods than the ROC curves. On average, for the five scRNA-seq datasets, GSVA was one of the top performers by ROC curve analysis and the top performer by PR curve analyses ( Figure 6A, B). GSVA’s performance was more robust than that of other methods in analyses where we subsampled genes from cell type signatures. All of these features are particularly important at this stage of the scRNA-seq field, as only limited information on cell type gene expression signatures is available. Notably, despite its relative simplicity, ORA showed a performance comparable to GSVA using most datasets and even higher using the liver dataset. A caveat of ORA is that it requires one extra step compared with other methods, which is to threshold the *Ě _xy_* matrix, typically using an arbitrary cutoff, often selected based on the overall distribution of gene expression values, as we used here. CIBERSORT and METANEIGHBOR were also comparable or even superior to GSVA in datasets where the number of cell clusters matched the number of cell types expected. For instance, both former methods outperformed GSVA using the PBMC-6-SeqWell datasets, and CIBERSORT’s performance was also higher than that of GSVA using the liver dataset. However, both CIBERSORT and METANEIGHBOR were markedly affected, and outperformed by GSVA, when the number of cell type signatures exceeded the number of cell clusters (i.e. ‘Tabula Muris 11’ and the PBCM-22-* datasets). A caveat of METANEIGHBOR is that in addition to the typical inputs (cell type signatures and *Ě _xy_* matrix) it requires a training phase based on known cell type gene markers to compute an AUC ROC as its prediction scores, but known cell type markers are not available for several scRNA-seq datasets. GSEA was the method with the lowest PR AUC values using all five datasets and was also one of the least stable in robustness analyses.

An interesting observation from the robustness analyses is that for some datasets and methods, subsamples of genes from cell type gene sets produced ROC and PR AUCs higher than those using 100% of the genes. This was particularly noticeable for CIBERSORT using retinal neurons, ‘Tabula Muris 11’, and the PBMC datasets, and for METANEIGHBOR using the PBMC datasets. This suggests that adding subsampling steps in the pipelines for some methods could improve their performance.

In terms of computing times, METANEIGHBOR was the fastest, and along with GSVA and ORA, offered implementations which were orders of magnitude faster than those of CIBERSORT and GSEA. Our results showed that CIBERSORT ‘binary’ performance was comparable to CIBERSORT ‘continuous’ by both ROC and PR curve analyses, and our implementation of the former reduced computing times between 1.3 and 6 times. Current publicly available scRNA-seq datasets typically contain on the order of thousands of cells, grouped into dozens of cell clusters. In our tests, each of the five tested methods completed the cell type prediction tasks in seconds or minutes. However, bigger datasets from the Human Cell Atlas ( Rozenblatt-Rosen *et al*., 2017) and other sources are expected to have millions of cells (e.g. 1.3 million brain cell from E18 mice, NCBI GEO: GSE93421) grouped into hundreds of clusters, for which the fastest method implementations will be preferred. Considering overall performance, robustness to incomplete cell type signatures, and computing times, we found that GSVA offers one of the best options to label cell clusters from scRNA-seq datasets.

A limitation of this study is that we included only five scRNA-seq datasets ( Table 1): liver, retinal neurons, Tabular Muris, and two PBMC datasets, plus variants of the latter three. This was due to the lack of reference cell type annotations needed for our ROC and PR curve analyses. As more scRNA-seq datasets become available and authors provide gold standard annotations of their cell types, our benchmark can be expanded. In the future, carefully annotated scRNA-seq cell clusters and their associated gene expression signatures and gene expression markers will likely replace literature curated gene expression marker sets, but we need many more and diverse scRNA-seq datasets to be generated to get to that stage. It would also be useful to identify recommended prediction score thresholds that maximize performance for each method as well as identify cell type gene sets that always perform poorly, but achieving general results from these analyses will likely need a larger and more diverse benchmark dataset. One way to address this is to predict cell types from individual cells, in which case a cross-validation approach can be used based on cluster labeling data ( Abdelaal *et al*., 2019), but this has the caveat that current generation scRNA-seq methods identify relatively few genes expressed per cell, compared to the cell clusters we analyzed here. Additionally, *k*-fold cross-validations can’t be applied to cell cluster level evaluations because a cluster represents a single object defined by a single vector capturing the average expression levels of all genes across all cells in the cluster.

Studying how cluster parameters and data structure (e.g. cluster density, fuzzy vs. hard clusters) affects our results should also be considered in future work. One of the challenges that we faced while adapting the LM22 signature to predict cell types in the scRNA-seq cell clusters generated by Zheng *et al*. (2017a) was that, even though both datasets correspond to PBMCs, the granularity of their cell type labels was different. For instance, the LM22 signature contains six T-cell types, including three CD4+ (naïve, memory resting, and memory activated), follicular helper, regulatory and gamma delta, whereas the dataset of Zheng *et al*. (2017a) contained labels for four T-cell related cell types: CD4+/CD25 T Regulatory, CD4+/CD45RO+ Memory, CD4+/CD45RA+/CD25- Naive T and CD4+ T Helper2. Thus, even though these two datasets both classify PBMCs, their cell types cannot be easily related one-to-one. This could be addressed with an ontology analogous to the Gene Ontology ( Ashburner *et al*., 2000) but dedicated to cell type annotations ( Bakken *et al*., 2017; Bard *et al*., 2005). Fortunately, the Cell Ontology is being developed for this purpose. This is particularly important as increasing numbers of signatures are expected to arise from initiatives like the Human Cell Atlas ( Rozenblatt-Rosen *et al*., 2017). However, it is an open question how cell cluster annotation performance will be affected when using these eventual comprehensive cell type gene expression marker set databases, as we observed that many methods are highly sensitive in precision-recall analysis when used with larger cell type marker gene set databases that contain additional cell types not represented in a given scRNA-seq dataset. Future work will need to study confusion matrices of all methods and better quantify precision scores. We hope our open source benchmark code can be extended as a useful starting point for future work.

## Data availability

### Underlying data

The datasets used in this study were processed from the following source data:

Single cell RNA-sequencing data from liver cells. Accession number, GSE115469. https://identifiers.org/geo/GSE115469.

Single cell RNA-sequencing of retinal bipolar cells. Accession number, GSE81905. https://identifiers.org/geo/GSE81905.

Single cell RNA-sequencing of Tabula Muris. Accession number, GSE109774. https://identifiers.org/geo/GSE109774.

Single cell RNA-sequencing data from peripheral blood mononuclear cells using 10X Chromium technology. Accession number, SRX1723926. https://identifiers.org/insdc.sra/SRX1723926.

Single cell RNA-sequencing data from peripheral blood mononuclear cells using Seq-Well technology. Accession number, GSE92495. https://identifiers.org/geo/GSE92495.

### Extended data

Zenodo: Supplementary data for “Evaluation of methods to assign cell type labels to cell clusters from single-cell RNA-sequencing data”. https://doi.org/10.5281/zenodo.2575049 version 2.1.1( Diaz-Mejia *et al*., 2019a).

This project contains the five processed scRNA-seq datasets—from liver cells ( MacParland *et al*., 2018), retinal neurons ( Shekhar *et al*., 2016b), Tabula Muris ( Tabula Muris Consortium *et al*., 2018a), peripheral blood mononuclear cells using 10X ( Zheng *et al*., 2017a) and Seq-Well ( Gierahn *et al*., 2017a)—examined in this study.

## Software availability

R and Perl scripts used to run and benchmark cell type labeling methods available from: https://github.com/jdime/scRNAseq_cell_cluster_labeling.

Archived code at the time of publication: https://doi.org/10.5281/zenodo.3350461 ( Diaz-Mejia *et al*., 2019b).

License: MIT license.
