# Supplementary material for: Evaluation of methods to assign cell type labels to cell clusters from single-cell RNA-sequencing data
Source: F1000Res. 2019 Oct 14;8:ISCB Comm J-296. Originally published 2019 Mar 15. [Version 3] doi: 10.12688/f1000research.18490.3 (PMC6720041; doi:10.12688/f1000research.18490.3)
Supplement: Peer review history

# Peer review: Reviewer response for version 3

**DOI:** 10.5256/f1000research.22823.r55093

**Published:** 2019-10-15

**Authors:** Saskia Freytag

**Affiliations:** 1 Epigenetics and Genomics, Harry Perkins Institute of Medical Research, Nedlands, WA, Australia

The authors have cleared all my concerns.

I confirm that I have read this submission and believe that I have an appropriate level of expertise to confirm that it is of an acceptable scientific standard.

# Peer review: Reviewer response for version 2

**DOI:** 10.5256/f1000research.22108.r52981

**Published:** 2019-09-02

**Authors:** Tallulah Andrews, Jimmy Tsz Hang Lee

**Affiliations:** 1 Wellcome Sanger Institute, Hixton, UK; 2 Wellcome Sanger Institute, Hinxton, UK

The authors have significantly improved the article and I believe it to be a valuable contribution to the literature. All my concerns have been addressed.

We confirm that we have read this submission and believe that we have an appropriate level of expertise to confirm that it is of an acceptable scientific standard.

# Peer review: Reviewer response for version 2

**DOI:** 10.5256/f1000research.22108.r52982

**Published:** 2019-08-30

**Authors:** Saskia Freytag

**Affiliations:** 1 Epigenetics and Genomics, Harry Perkins Institute of Medical Research, Nedlands, WA, Australia

The authors have addressed most of my concerns, however I still do not fully understand why other specialized approaches for assigning labels to single cells. In fact there has been a recent publication in bioRvix of automatic single cell identification methods for single cell RNA-sequencing data ^1^. I think it would be important to clarify this and acknowledge this paper.

I confirm that I have read this submission and believe that I have an appropriate level of expertise to confirm that it is of an acceptable scientific standard, however I have significant reservations, as outlined above.

## References

[1] : A comparison of automatic cell identification methods for single-cell RNA-sequencing data. *bioRxiv*.2019; 10.1101/644435 10.1101/644435 PMC673428631500660

# Response

**Published:** 2019-10-07

**Authors:** J. Javier Diaz-Mejia

**Affiliations:** University Health Network, Canada, Canada

*R1-Q1) The authors have addressed most of my concerns, however I still do not fully understand why other specialized approaches for assigning labels to single cells. In fact there has been a recent publication in bioRvix of automatic single cell identification methods for single cell RNA-sequencing data1. I think it would be important to clarify this and acknowledge this paper.*

*References*

*1. Abdelaal T, Michielsen L, Cats D, Hoogduin D, Mei H, Reinders M, Mahfouz A: A comparison of automatic cell identification methods for single-cell RNA-sequencing data. bioRxiv. 2019. Publisher Full Text*

R1-A1) We had previously cited this paper in our discussion section (that paper is now published in Genome Biology). Our evaluation results are complementary. The main difference is that Abdelaal *et al*. evaluate methods that assign cell type labels to each single cell in a data set while we evaluate methods that assign cell type labels to clusters of similar cells. While these use cases seem similar enough, methods designed for one case cannot be easily run on the other case and the evaluation methods of Abdelaal *et al*. and our paper are fundamentally different and incompatible. In particular, Abdelaal *et al*. use a cross-validation approach where they split a data set into two sets of single cells, then apply the methods to both and compare the results. For example, if there are 1000 cells in a scRNA-seq dataset, Abdelaal *et al*. (2019) would split the data into 200 cell parts, then for a given fold, they would take 80% of the data (800 cells) to train each method and test (annotate) the remaining 200 cells; and compare the 200 cell annotations against the labels provided by the authors of the original scRNA-seq dataset. It is not possible to apply this method at the cell cluster level because a cluster represents a single object defined by a single vector capturing the average expression levels of all genes across all cells in the cluster. We also can’t change our clusters in any way because they have been expertly defined and annotated to specific cell types - any change will invalidate this expert annotation. These differences evaluating cell cluster vs. individual cell methods are the reason why it was challenging to incorporate MetaNeighbor into our analysis, requiring source code modifications and extensive communication with the tool authors. Ultimately, the field will be well served by having two complementary benchmark papers, with Abdelaal *et al*. focusing on individual cell classification methods and ours focusing on cluster classification methods. We have clarified this in our updated manuscript.

# Peer review: Reviewer response for version 1

**DOI:** 10.5256/f1000research.20232.r45813

**Published:** 2019-04-01

**Authors:** Lindsay Cowell

**Affiliations:** 1 Department of Population and Data Sciences, University of Texas Southwestern Medical Center, Dallas, TX, USA

The authors address an important problem, which is the need for systematic and reproducible approaches for assigning cell type labels based on single cell transcriptome data. They use three data sets with gold standard cell type annotations available and compare the performance of four computational tools on these data sets. The authors measure performance using ROC curves and plots of precision versus recall. They also assess performance over subsamples of the data used as reference gene expression patterns for cell types (either cell type-specific gene sets or cell type-specific expression profiles). In general, they found that all four methods perform reasonably well, although ORA and GSVA perform more consistently well across the three data sets. I do have some questions about the details of how the work was done. The answers to these questions are important for interpreting the results, reproducing the work, or extending it to include additional tools.

1. Presumably the approach to creating the cell clusters, and how dense versus diffuse the clusters are, can have an impact on performance and confidence in the output?
2. How exactly were clusters mapped to cell types? From Figure 1E, it appears that each of the four tools generates a numerical vector for each cell type that contains a score for each cluster, presumably corresponding to the likelihood that that cluster is of the corresponding cell type.
  - Is a cluster always assigned to the cell type corresponding to its highest score? (presumably yes).
  - In the example, each cell type and each cluster has only a single high score with all other scores being very small. What is the distribution of scores typically? Do clusters sometimes have multiple high scores? Were ties ever observed?
  - Can multiple clusters map to the same cell type?
  - Must a cluster be assigned to a cell type? Or could some remain unassigned?
3. How were the performance curves generated? What parameter was varied?

I confirm that I have read this submission and believe that I have an appropriate level of expertise to confirm that it is of an acceptable scientific standard, however I have significant reservations, as outlined above.

# Response

**Published:** 2019-08-17

**Authors:** J. Javier Diaz-Mejia

**Affiliations:** University Health Network, Canada, Canada

***R3-Q1)** Presumably the approach to creating the cell clusters, and how dense versus diffuse the clusters are, can have an impact on performance and confidence in the output?*

**R3-A1)** We agree that cluster density and other structure in the data will likely impact automatic cluster annotation performance. Investigating the relationship between a given structure in the data (e.g. density vs. sparseness) and performance would require simulations that may not be realistic. Thus, we limited our analysis to published data with available gold standards. We have now added this point to the discussion.

***R3-Q2)***

*[a] How exactly were clusters mapped to cell types? From Figure 1E, it appears that each of the four tools generates a numerical vector for each cell type that contains a score for each cluster, presumably corresponding to the likelihood that that cluster is of the corresponding cell type.*

*[b] Is a cluster always assigned to the cell type corresponding to its highest score? (presumably yes).*

*[c] In the example, each cell type and each cluster has only a single high score with all other scores being very small. What is the distribution of scores typically? Do clusters sometimes have multiple high scores? Were ties ever observed?*

*[d] Can multiple clusters map to the same cell type?*

*[e] Must a cluster be assigned to a cell type? Or could some remain unassigned?*

**R3-A2)**

[a] Correct, each tool generates a numerical vector as the reviewer describes.

[b] Yes, a cluster is always assigned to the cell type corresponding to its highest score.

[c] In the methods that we compared, each cell cluster vs. each cell type receives only one score. As can be observed in our new Figure 6E, most cell clusters which were incorrectly classified (i.e. that were not the top-1 ranked prediction) still had top-ranks (ticker distribution in the violin plots closer to the top-1 ranks), which indicates that some clusters can have multiple high scores.  We found that 118 out of all 1,276 (9.2%) cell cluster labeling predictions we ran showed ties in the top-score: 65 of the 118 ties (68%) corresponded to METANEIGHBOR ‘binary, 24 (20%) to ORA, 15 (13%) to METANEIGHBOR ‘continuous’, 10 (8%) to GSEA, and 4 (3%) to GSVA. None of the CIBERSORT analyses showed ties.

[d] Yes, multiple clusters can map to the same cell type and this is particularly the case for the newly incorporated Tabula Muris dataset, where 130 cell clusters map to 53 cell types. This doesn’t affect our evaluation because a method is not penalized for predicting that multiple clusters have the same cell type annotation.

[e] Yes, a cluster must be assigned a cell type in our case because all clusters have a cell type assignment in our gold standards. In the case of the newly incorporated PBMC-SeqWell data (Gierahn *et al*., 2017), some of the cell clusters were labeled as ‘Removed_’ by the authors, and they didn’t classify those clusters into cell types, thus we did not include these in our analysis.

As mentioned above in response to reviewers 1 and 2, we’ve updated the Methods section “Implementation of tested methods and transformation of enrichment metrics for ROC and PR analyses” to clarify all of these points.

***R3-Q3)** How were the performance curves generated? What parameter was varied?*

**R3-A3)** As mentioned above in response to reviewers 1 and 2, we’ve updated the Methods section “Implementation of tested methods and transformation of enrichment metrics for ROC and PR analyses” to clarify this. For each dataset, we combine all cell type gene set prediction scores for a method across all clusters into one column and vary the prediction score threshold to compute the ROC and PR curves.

# Peer review: Reviewer response for version 1

**DOI:** 10.5256/f1000research.20232.r45811

**Published:** 2019-03-22

**Authors:** Tallulah Andrews, Jimmy Tsz Hang Lee

**Affiliations:** 1 Wellcome Sanger Institute, Hixton, UK; 2 Wellcome Sanger Institute, Hinxton, UK

Diaz-Mejia et al. test the ability of four different algorithms to correctly annotate a set of clusters identified in single-cell RNA-seq data. They find that GSVA tends to be the most accurate and fastest method, interestingly they find ORA and GSVA are much more robust to small numbers of marker genes than GSEA or CIBERSORT. This is a very useful and timely study, as manual annotation of cell-types is currently the main bottleneck when analyzing single-cell RNA-seq data.

 Comments:

1. It was unclear to me how the accuracy of the classification methods was evaluated. What was the gold standard truth used for each dataset? Were clusters assigned to (a) the single cell-type for which they had the greatest score or (b) all cell-types where their score exceeded some threshold, or (c) to the single cell-type for which they had the greatest score provided that score was above some threshold or another approach? This is crucial to interpreting the PR and ROC curves presented in the results.
  Based on the first sentence of the “Precision-Recall curve analysis” section: I inferred you to be using method (c), but using such a method should not necessarily lead to recall values of 1 as clusters which are more similar to an incorrect cell-type than to the correct cell-type would never become true positives. Thus, I had inferred you to be using method (b) based on Figure 2. It would be very helpful to add a section to the Methods explaining precisely how the accuracy was evaluated.
2. In addition, I suggest adding figures/tables for the accuracy of each classification approach (% of clusters correctly assigned) when all clusters are simply assigned to the cell-type for which they have the highest score, since I expect this to be the most common approach users of these classifications would take.
3. The main weakness of the paper, as the authors admit, is the small number of datasets used to test the classification methods, particularly since the variability in performance between datasets was high. It would be useful to show reproducibility of the results in additional datasets.
  We acknowledge identifying marker gene lists for many different tissues can be very time consuming, there are datasets similar to those the authors have already have markers for that they could use. E.g. mouse retina: Shekhar et al. 2016 ^1^, PBMCs: Gierahn et al. 2017 (Seq-Well) ^2^. Alternatively, they could do cross-comparisons using the two mouse cell atlas (Tabula Muris ^3^ and Mouse Cell Atlas ^4^). Or use datasets such as Pollen et al., 2014 ^5^ where gold-standard cell-type identity is known by design.
4. The authors show that performance degrades when small numbers of marker genes are used by the classifiers. Is it the case that more marker genes is always better or does performance also degrade if too many genes are used?

We confirm that we have read this submission and believe that we have an appropriate level of expertise to confirm that it is of an acceptable scientific standard, however we have significant reservations, as outlined above.

## References

[1] : Comprehensive Classification of Retinal Bipolar Neurons by Single-Cell Transcriptomics. *Cell*.2016;166(5) : 10.1016/j.cell.2016.07.054 1308-1323.e30 10.1016/j.cell.2016.07.054 27565351PMC5003425

[2] : Seq-Well: portable, low-cost RNA sequencing of single cells at high throughput. *Nat Methods*.2017;14(4) : 10.1038/nmeth.4179 395-398 10.1038/nmeth.4179 28192419PMC5376227

[3] : Single-cell transcriptomics of 20 mouse organs creates a Tabula Muris. *Nature*.562(7727) : 10.1038/s41586-018-0590-4 367-372 10.1038/s41586-018-0590-4 30283141PMC6642641

[4] : Mapping the Mouse Cell Atlas by Microwell-Seq. *Cell*.2018;172(5) : 10.1016/j.cell.2018.02.001 1091-1107.e17 10.1016/j.cell.2018.02.001 29474909

[5] : Low-coverage single-cell mRNA sequencing reveals cellular heterogeneity and activated signaling pathways in developing cerebral cortex. *Nat Biotechnol*.2014;32(10) : 10.1038/nbt.2967 1053-8 10.1038/nbt.2967 25086649PMC4191988

# Response

**Published:** 2019-08-17

**Authors:** J. Javier Diaz-Mejia

**Affiliations:** University Health Network, Canada, Canada

***R2-Q1) **It was unclear to me how the accuracy of the classification methods was evaluated. What was the gold standard truth used for each dataset? Were clusters assigned to (a) the single cell-type for which they had the greatest score or (b) all cell-types where their score exceeded some threshold, or (c) to the single cell-type for which they had the greatest score provided that score was above some threshold or another approach? This is crucial to interpreting the PR and ROC curves presented in the results.*

*Based on the first sentence of the “Precision-Recall curve analysis” section: I inferred you to be using method (c), but using such a method should not necessarily lead to recall values of 1 as clusters which are more similar to an incorrect cell-type than to the correct cell-type would never become true positives. Thus, I had inferred you to be using method (b) based on Figure 2. It would be very helpful to add a section to the Methods explaining precisely how the accuracy was evaluated.*

**R2-A1) **Apologies for the confusion around this point. We have now clarified how the ROC and PR curves were computed in Figure 1 and the text, as described for reviewer 1, above. We combine all cell type gene set prediction scores for a method across all clusters into one column and vary the prediction score threshold to compute the ROC and PR curves. A cluster is only allowed to be correctly labeled using one cell type, as enforced by our gold standard cluster annotation data (the set of cell types an author used to label their given cell clusters). So this matches strategy (c).

***R2-Q2) **In addition, I suggest adding figures/tables for the accuracy of each classification approach (% of clusters correctly assigned) when all clusters are simply assigned to the cell-type for which they have the highest score, since I expect this to be the most common approach users of these classifications would take.*

**R2-A2) **Percent of clusters correctly assigned is now included in Figure 6C and Supplementary Table 1. It is useful to have a range of performance indicators to capture different performance facets.

***R2-Q3) **The main weakness of the paper, as the authors admit, is the small number of datasets used to test the classification methods, particularly since the variability in performance between datasets was high. It would be useful to show reproducibility of the results in additional datasets.*

*We acknowledge identifying marker gene lists for many different tissues can be very time consuming, there are datasets similar to those the authors have already have markers for that they could use. E.g. mouse retina: Shekhar et al. 20161, PBMCs: Gierahn et al. 2017 (Seq-Well)2. Alternatively, they could do cross-comparisons using the two mouse cell atlas (Tabula Muris3 and Mouse Cell Atlas4). Or use datasets such as Pollen et al., 20145 where gold-standard cell-type identity is known by design.*

**R2-A3) **We thank the reviewer for suggesting these datasets. We already used Shekhar *et al*. 2016 in version 1 of our paper. In the current version, we added Gierahn *et al*. (2017) as the authors provide cell type labels for the cell clusters, and used the LM22 cell type signatures as input for the prediction methods. We also added the Tabula Muris dataset. We contacted one of the Tabula Muris authors (Angela Pisco), who kindly gave us access to a set of cell type signatures curated by experts on tissues of the Tabula Muris dataset. From the 20 tissues provided in the Tabula Muris data we could map 11 of them into the dataset of cell type signatures and that is what we used as ‘Tabula Muris 11’ in the current version of our paper. We also investigated the influence of cell type signatures using the PBMC datasets (10X and Seq-Well) using either the full LM22 signature database, that we call ‘PBMC-22’, or only the six cell types expected to occur in the data, that we call ‘PBMC-6’. Altogether, we provide analysis using eight dataset variants, up from the three in our initial manuscript.

***R2-Q4) **The authors show that performance degrades when small numbers of marker genes are used by the classifiers. Is it the case that more marker genes is always better or does performance also degrade if too many genes are used?*

**R2-A4)** In general, having more marker genes is better, but not always. We approached this question by examining the influence of the number of genes in each gene set (x-axis) and asking what rank does the corresponding gold standard positive receive (y-axis). As can be seen in Figure 6E, the most common scenario is that the fewer the number of genes in the signatures, the more chances that the prediction is incorrect (i.e. assigned a rank lower than the top-rank). However, there are a few exceptions, like ORA in the 11-20 genes bin, where we found more incorrect predictions than having 6-10 genes, or CIBERSORT, which had higher error rate in the 31-50 genes category, than in the 11-20 or 21-30 categories. Thus, it is possible to use too many genes, but it is not always clear how many genes this will be and the performance drop is not great for most of the cases we have data for. We have added this analysis to the paper.

# Peer review: Reviewer response for version 1

**DOI:** 10.5256/f1000research.20232.r45814

**Published:** 2019-03-20

**Authors:** Saskia Freytag

**Affiliations:** 1 Epigenetics and Genomics, Harry Perkins Institute of Medical Research, Nedlands, WA, Australia

Diaz-Mejia et al have produced a nice research article on assessing methods for assigning cluster labels to cell clusters from scRNA-seq. I think this work is of great importance, but I felt that some crucial cluster labelling methods were not compared. I hope the authors can update the article with some of the suggestions:

  

- The biggest suggestion for improvement is the choice of methods that the authors compare. The authors chose to adapt 4 methods originally developed for bulk RNA-seq in order to label clusters. While their approach is commendable, none of their adapted methods reflect the current standard practice in the field. Additionally, their claim that methods for cluster labelling in scRNA-seq are too immature or implemented as web-servers is not true. The website scRNA-tools.org lists 29 methods in this category. Many of these methods, such as scMCA, MetaNeighbour and scmap, are well-established and frequently used in the field. Furthermore, many of these methods recommend using annotated scRNA-seq datasets as references instead of bulk data. Hence, it would be great if the authors could include some of these tools in their analysis.

- I am confused as to which classifier parameter was varied in order to generate the ROCs. Were these comparable across the different methods?

- It would be interesting to see what the effect of varying the cluster resolution is to the ability of the methods to accurately label the populations. Do you obtain more diverse labelling when there are more clusters?

- LM22 is a great reference dataset, but recently a new dataset has become openly accessible. This dataset, generated by Monaco et al ^1^, characterizes 29 human immune cell types by RNA-seq and flow cytometry. It would be interesting to see if the use of this dataset leads to an improvement.

- I think it would be helpful for the reader if the authors could summarize their results. The sheer number of comparisons made, means that the reader can feel overwhelmed at the end. A figure summarizing the various results for each method in each dataset could help clarify the message.

 

 Thank you for making your code publicly available.

I confirm that I have read this submission and believe that I have an appropriate level of expertise to confirm that it is of an acceptable scientific standard, however I have significant reservations, as outlined above.

## References

[1] : RNA-Seq Signatures Normalized by mRNA Abundance Allow Absolute Deconvolution of Human Immune Cell Types. *Cell Rep*.2019;26(6) : 10.1016/j.celrep.2019.01.041 1627-1640.e7 10.1016/j.celrep.2019.01.041 30726743PMC6367568

# Response

**Published:** 2019-08-17

**Authors:** J. Javier Diaz-Mejia

**Affiliations:** University Health Network, Canada, Canada

***R1-Q1)** The biggest suggestion for improvement is the choice of methods that the authors compare. The authors chose to adapt 4 methods originally developed for bulk RNA-seq in order to label clusters. While their approach is commendable, none of their adapted methods reflect the current standard practice in the field. Additionally, their claim that methods for cluster labelling in scRNA-seq are too immature or implemented as web-servers is not true. The website scRNA-tools.org lists 29 methods in this category. Many of these methods, such as scMCA, MetaNeighbour and scmap, are well-established and frequently used in the field. Furthermore, many of these methods recommend using annotated scRNA-seq datasets as references instead of bulk data. Hence, it would be great if the authors could include some of these tools in their analysis.*

**R1-A1)** We thank the reviewer for their comments. We have added MetaNeighbor to the methods compared. The implementation of MetaNeighbor required considerable communication with one of the method developers (M. Crow) who kindly guided us on which parts of the MetaNeighbor source code we needed to modify to make one of its variants (MetaNeighborUS) compatible with the type of task in our study. As we detail in the Methods section ‘Implementation of tested methods and transformation of enrichment metrics for ROC and PR analyses’, the original goal of MetaNeighbor is to quantify cell type replicability across  scRNA-seq datasets (that the authors call ‘studies’); whereas in our comparison, we are “comparing” all the clusters in one scRNA-seq dataset against known cell type specific gene sets or gene expression profiles. Similar to MetaNeighbor, Scmap projects cells from a scRNA-seq experiment on to the cell types or individual cells identified in a different experiment. We would need to apply similar workarounds and modify its code to use it in our study. Although we acknowledge that adding more methods to our comparison would make our results more complete, these other methods were not designed for the specific task we evaluate and would require code modifications to work on our input data. However, we provide an extensible framework, code and datasets that others can use for additional benchmarks. We now clarify this point in the paper.

***R1-Q2)** I am confused as to which classifier parameter was varied in order to generate the ROCs. Were these comparable across the different methods?*

**R1-A2) **Sorry for the confusion. For a set of cell clusters, a given method was used to score each cluster against all cell type gene sets resulting in a matrix of cell type prediction scores per cluster. All scores in this matrix were combined into one column to capture all cell type prediction scores across all clusters and this set of prediction scores was varied to generate the ROC and PR curves. This is now clarified in Figure 1 and the text.

***R1-Q3)** It would be interesting to see what the effect of varying the cluster resolution is to the ability of the methods to accurately label the populations. Do you obtain more diverse labelling when there are more clusters?*

**R1-A3) **Presumably yes. However, there is a methodological barrier that prevents us from investigating this aspect of the data using our current evaluation design. Authors of the analyzed datasets provided gold standard annotations only at a single resolution per dataset, and we use these. Reclustering the original data to test other resolutions would require gold standards to be created for those resolutions (ideally by the original authors). However, we agree with the reviewer that studying the influence of cell cluster resolution is an interesting question. As the field moves towards increasing the number of scRNA-seq datasets annotated following standard ontology-based cell type annotations that consider a hierarchy of cell types at multiple granularities, this question could be addressed. We have added this to our discussion.

***R1-Q4) **LM22 is a great reference dataset, but recently a new dataset has become openly accessible. This dataset, generated by Monaco et al1, characterizes 29 human immune cell types by RNA-seq and flow cytometry. It would be interesting to see if the use of this dataset leads to an improvement.*

**R1-A4) **Thanks for the pointer. We decided to keep the LM22 dataset because only six of the 22 cell types represented in it could be mapped into the PBMC data we analyzed. The Monaco dataset does not improve this number. Only five of the 17 cell types represented in the Monaco signature for RNA seq data are present in the PBMC data we analyzed. Furthermore, the ROC AUC and PR AUC values obtained using the LM22 and the Monaco signature are comparable to each other (Supplementary Table 2).

***R1-Q5)** I think it would be helpful for the reader if the authors could summarize their results. The sheer number of comparisons made, means that the reader can feel overwhelmed at the end. A figure summarizing the various results for each method in each dataset could help clarify the message.*

**R1-A5) **Thanks for raising this point. We have now included summary Figure 6.

***R1-Q6) **Thank you for making your code publicly available.*

**R1-A6) **Thanks. We have updated our GitHub repository with the MetaNeighbor implementation and modifications to our main wrapper to make it easier to incorporate new methods.
